# Complete genome of *Rhodococcus pyridinivorans* FL1 isolated from malodorous water, a bacterium capable of degrading aniline

**DOI:** 10.1128/mra.00241-25

**Published:** 2025-06-09

**Authors:** Xiaoli Fang, Hongfei Wang, Jingxuan Deng, Jian Gao, Mingjun Liao

**Affiliations:** 1 Hubei Key Laboratory of Environmental Geotechnology and Ecological Remediation for Lake & River, Hubei University of Technology 47896, Wuhan, Hubei, China; 2 Hengzhen Wuxi Biotechnology Co, Ltd, Wuxi, China; Montana State University, Bozeman, Montana, USA

**Keywords:** *Rhodococcus pyridinivorans*, aniline degradation

## Abstract

A novel *Rhodococcus pyridinivorans* strain FL1, capable of degrading aniline, was isolated from an urban river ecosystem. The complete genome sequence of this strain was presented, and the key enzymes involved in the aniline degradation pathway, including catechol 1,2-dioxygenase (*catA*) and 3-carboxy-*cis*,*cis*-muconate cycloisomerase, were systematically annotated‌.

## ANNOUNCEMENT

Industrial wastewater from textile dyeing, pharmaceutical, and chemical manufacturing sectors typically contains a certain concentration of aniline derivatives (1). Bioaugmentation strategies employing aniline-degrading microbial strains have been demonstrated as a cost-effective way to eliminate this inhibition (2)‌. *R. pyridinivorans* FL1 was isolated from an urban river in Zhongshan City, China. Analysis of the 16S rRNA gene (NCBI: PV476698.1) revealed 99.93% sequence identity with *R. pyridinivorans* SS2 (NCBI: KY082045.1). The Query Cover was 98%.

*R. pyridinivorans* FL1 was cultured in MSM containing 500 mg/L aniline at 30°C with 150 rpm shaking (3). Genomic DNA was extracted using the Wizard HMW DNA Purification Kit (Promega, USA) and quantified using a TBS-380 fluorometer (Turner BioSystems Inc., Sunnyvale, CA). Genome sequencing was conducted using a combination of PacBio RS II single-molecule real-time (SMRT) sequencing and the Illumina sequencing platform. For Illumina sequencing, 1 µg of genomic DNA was fragmented using Covaris M220, and libraries were prepared using the NEXTflex Rapid DNA-Seq Kit (Bioo Scientific, USA). Paired-end sequencing (2 × 150 bp) was performed on the NovaSeq/HiSeq Xten platform (Illumina, USA). For PacBio sequencing, the genomic DNA was fragmented into 10 kb fragments, and 15 µg of genomic DNA was processed using G-tubes (Covaris, MA) for single-molecule library construction, followed by sequencing on the PacBio RS II platform. PacBio yielded 81,082 reads (757,248,223 bp), and Illumina 4,156,895 * 2 raw paired-end reads (1,255,382,290 bp), with a depth of 139.45. No PacBio quality control was done; fastp (v0.23.0) trimmed Illumina data. SOAPdenovo (v2.04) (4) was used for the assembly of Illumina data, while Canu (v2.1.1) and Unicycler (v0.4.8) software were employed for PacBio data assembly (5). Reads were assembled into contigs, and then the genome was automatically circularized with the tool nextdenovo (v2.5.2) to obtain complete chromosomal and plasmid genomes. Illumina assembly length was 5,794,977 bp (N50: 147,962 bp), and PacBio was 5,430,293 bp (N50: 4,882,508 bp). The coverage rates of Illumina and PacBio read mapping are 95.52% and 99.4%, respectively.

The complete genome of *R. pyridinivorans* FL1 consists of a single chromosome (4,882,508 bp), two large plasmids (284,626 bp and 156,891 bp), and two small plasmids (54,091 bp and 52,177 bp), with a GC content of 67.72%. A total of 5,115 CDS, 54 tRNAs, and 12 rRNAs were predicted using Prodigal (v2.6.3). The average nucleotide identity (ANI) with *R. pyridinivorans* SB3094 (NCBI: CP006996.1) was 98.8%, as calculated using the ANI Calculator (6).

Functional annotation using Diamond (v0.9.25) (7) against the Swiss-Prot database identified the cytochrome P450 monooxygenase gene (CYP107DY1) (8), suggesting that the strain may catalyze the hydroxylation of aniline through this enzymatic system. Annotation against the NR database revealed genes encoding catechol 1,2-dioxygenase (*catA*) and 3-carboxy-*cis*,*cis*-muconate cycloisomerase, which are involved in the cleavage of the aromatic ring of ortho-aminophenol (9), producing the key intermediate metabolite 2-aminomuconic semialdehyde. The cycloisomerase catalyzes the intramolecular cyclization of this semialdehyde to form muconolactone (10), which ultimately enters the tricarboxylic acid (TCA) cycle for complete metabolism.

## Data Availability

The nucleotide sequence of R. pyridinivorans FL1 has been deposited in the NCBI database under the BioProject accession number PRJNA1226984. The raw sequencing reads are available under the Sequence Read Archive (SRA) accession number SRR32498772. The complete chromosome sequence is accessible in GenBank under the accession number CP182829.1, while the four plasmid sequences are deposited under the following GenBank accession numbers: Plasmid A: CP182830.1, Plasmid B: CP182831.1, Plasmid C: CP182832.1, and Plasmid D: CP182833.1.
